# Reflective Practice: A Method to Improve Teachers’ Well-Being. A Longitudinal Training in Early Childhood Education and Care Centers

**DOI:** 10.3389/fpsyg.2019.02574

**Published:** 2019-11-26

**Authors:** Ada Cigala, Elena Venturelli, Martina Bassetti

**Affiliations:** ^1^Department of Humanities, Social Sciences and Cultural Industries, University of Parma, Parma, Italy; ^2^PRO.GES. TRENTO - s.c.s. Cooperativa Sociale, ONLUS, Trento, Italy

**Keywords:** well-being, preschool teachers, longitudinal training, self-efficacy, sense of belonging, agency, multi-age classroom, reflective practice

## Abstract

Various studies focused on educational contexts (0–6 years) point out that early childhood multi-age classrooms provide better learning strategies and socio-emotional competences of children, compared to single-grade classrooms. However, these studies have also shed light on the significant role of teachers. The multi-age classroom in particular is an opportunity for child development, provided that teachers consider problem-solving, flexibility, and co-construction as effective education strategies. Starting from these reflections, this study aimed to verify the efficacy of longitudinal training for the purpose of advancing the perceived well-being of early childhood teachers of multi-age groupings (18–54 months). Eight teachers and one pedagogical coordinator of an Italian Early Childhood Education and Care center took part in the study. All the participants were females. The critical aspect identified by the teachers was the multi-age classroom, which was perceived as making teaching and learning very difficult and ineffective for both themselves and for the children. The training lasted 10 months and implied a methodology focused on *observations* of some activities and *reflective practice* in the group that concerned both teachers and the pedagogical coordinator. The training involved the drafting of two types of written protocols: the observational reports of the specific activities observed (20), and the descriptive reports of reflective sessions (6). The content analysis of the reports revealed various and interesting themes regarding the teachers’ perceived well-being, in terms of thoughts, behaviors, and feelings. The qualitative and longitudinal analysis of the themes that emerged in these protocols highlighted different processes of change in the teachers’ perception, in particular with respect to three specific dimensions of well-being: *sense of belonging*, *self-efficacy*, and *agency*. At the end of the training, the teachers experienced a greater sense of belonging to the group of colleagues, a greater sense of self-efficacy, and an idea of themselves as active and meaningful participants. These results supported different reflections regarding the modalities through which to enhance the perceived well-being of teachers.

## Introduction

In recent years from many sides, a number of efforts have emerged to increase children’s access to high-quality Early Childcare and Education Centers (ECEC), but various authors argue that some attention and study must be given also to the teachers and to their workplaces, with particular focus on their perceived well-being in these contexts (Roffey, 2012; Zinsser and Zinsser, 2016). Indeed, some recent studies have pointed out that many preschool teachers report serious psychological health problems (e.g., Whitaker et al., 2013). It is indeed stressful and tiring work both from a physical and psychological point of view, having to deal with the care and education of very young children, some even just a few months old.

Moreover, the literature on the teachers’ well-being in the school context, and in particular in the Early Childcare and Education Centers, shows a rather fragmented framework of data and empirical models (Hall-Kenyon et al., 2014; De Stasio et al., 2017; Benevene et al., 2018). The studies specifically show that the well-being construct has been described in different terms by different authors (Bowling et al., 2010; Hall-Kenyon et al., 2014). It can be noted that most of the research had focused on the individual dimensions that contribute to the teachers’ well-being, such as emotional competence, temperament, self-esteem, educational qualifications, and years of professional experience (Hall-Kenyon et al., 2014; De Stasio et al., 2017; Benevene et al., 2018).

Fewer authors have proposed to analyze teachers’ well-being addressing the social or systemic dimensions. In the theoretical framework of these studies, teachers’ well-being is conceptualized as an emerging quality of a complex of interactions between various factors, such as relationships with children, between colleagues, with managers, and with parents, as well as the interactions with the elements of the space of the school context (Holmes, 2005; Critchley and Gibbs, 2012; Roffey, 2012;Zinsser and Zinsser, 2016).

These studies, albeit not numerous, appear to be very interesting, as they have a high ecological value (Roffey, 2012); central to these contributions is the concept of “*workplace environment*” that includes different aspects (spatial, psychological, and behavioral) interacting reciprocally and taking on several configurations (Zinsser and Zinsser, 2016).

An in-depth analysis of these studies allows us to highlight how the authors in the various studies focused on different aspects of the workplace, proposing specific and different constructs. For example, some studies have shown that there is a close positive relationship between the teachers’ well-being and the perception of *internal social support*, defined as the possibility for teachers to experiment with spaces of constructive sharing between colleagues and with the manager that are aimed to identify functional strategies to deal with complex and difficult professional situations (Halbesleben, 2006; Karademas, 2006; Sanglim and Sungeun, 2016).

In other studies, instead, the concept of *psychological safety of the environment* was introduced, understood as the perception of the work group as a context in which there is respect, acceptance, care, and mutual professional trust. On a personal level, a school system perceived as psychologically secure gives teachers the experience of feeling valued, respected, and cared for (Edmondson, 1999; Dollard and Bakker, 2010; Roffey, 2012; Edmondson and Lei, 2014).

According to Zinsser and Zinsser (2016), “One critical component of the supportive workplace climates is the construction of a workplace environment that teachers perceive as psychologically safe and in which they feel capable of engaging in the challenging work of early childhood education” (p. 49).

Still within the research overview on the teachers’ well-being, other authors have introduced the concept of *sense of belonging* to the school or, in particular, to the group of colleagues, which has been declined as the perception of having a precise role within the system, a role that is also recognized by other group members. On a personal level, the sense of belonging for an individual corresponds to feeling acknowledged, valued, and included (Rowe et al., 2007; Wike and Fraser, 2009; Skaalvik and Skaalvik, 2010; Roffey, 2012).

This sense of belonging, expressed by some authors as *connectedness* (Rowe et al., 2007) or *sense of community* (McGinty et al., 2008; Hall-Kenyon et al., 2014), is deeply related to the quality of interactions, in particular when the teachers in the group of colleagues feel that trust and reciprocity predominate, that they are positively connected with each other, and that there is a greater chance to perceive well-being.

Finally, still within the relational-systemic approach to the study of the teachers’ well-being, the construct of *agency* appears to be extremely relevant. The perceived agency refers to the perception by teachers of the possibility to participate in organizational decision-making (Sanglim and Sungeun, 2016) and to have an active role within the school system, a role that is recognized by the whole group of colleagues (Wilson and Deaney, 2010; Priestley et al., 2013; Hadar and Benish-Weisman, 2019). Specifically, the agentic capacity refers to the concept that teachers do not simply repeat given practices, rather, they have a capacity for pro-active and autonomous actions, that favor important transformations and changes both in themselves and in the whole workplace environment (Hadar and Benish-Weisman, 2019). For Wilson and Deaney (2010), agency can be considered a combination of intention and action that influences experience. Several studies have investigated the relationship between the teachers’ perceived agency and the perception of their well-being within the workplace, highlighting interesting connections (Helgeson, 1994; Buchanan and Bardi, 2015; Hadar and Benish-Weisman, 2019).

In all these studies, the authors emphasized the need to orient future research toward teachers’ well-being in a more ecological direction, favoring *qualitative*, *situational*, and *longitudinal methodologies,* which allow us to analyze the processual and interactive dimensions of teachers’ well-being. These indications address the research toward studies focused on specific situations considered particularly challenging from the point of view of the school system; in this sense, the case studies seem to be a suitable method (Critchley and Gibbs, 2012; Roffey, 2012; Cook et al., 2016; Hadar and Benish-Weisman, 2019; Heyeres et al., 2019).

In accordance with this perspective, the present research-intervention was designed to study the well-being of preschooler teachers, conceptualized, not so much as a construct in general, but rather as well-being perceived in a specific situation considered critical and problematic from the teachers’ point of view. In particular, the study involves pre-school teachers engaged in the management of a multi-age group (18–54 months) that include children from two different classes.

It is important to underline that the incentive to create multi-age classes is part of a general orientation in Italian Early Childcare and Education Centers toward the construction of an integrated 0–6 years system. The Italian childcare services are today mostly structured according to a split 0–3/3–6 years system, but recently, there has been a move toward the construction of an integrated system according to a recent law (Law no. 107/2015, art. 1, paragraph 181, letter e; legislative decrees 2017).

The literature on multi-age classrooms in the pre-school age highlights interesting aspects (Maeda, 1994; Veenman, 1996; Dersheid, 1997; McClellan and Kinsey, 1999; Aina, 2001; Gerard, 2005; Logue, 2006; Quann and Wien, 2006; Edwards et al., 2009).

Among the various studies on the topic, the contribution of Edwards et al. (2009) appears to be very interesting; it is a longitudinal study conducted in the preschool educational services in Australia for a period of 11 months. In this study, the teachers’ point of view with respect to the multi-age classes was surveyed by analyzing their ideas, their speech, and their practices in the workplace context.

In general, the results of these studies show that multi-age classes foster children’s cognitive and social learning. In particular, the multi-age classes are contexts that promote language acquisition, problem-solving skills, active participation in the learning process (Maeda, 1994; Chapman, 1995), the positive management of peer relations, positive conflict resolution, and greater attention to individual differences both by teachers and children (Aina, 2001; Baumgartner and Bombi, 2005; Edwards et al., 2009).

But the most interesting aspect that, in different ways, seems to emerge from the research, is that in the preschool period the multi-age class context fosters child development only if the teachers share some aspects of the educational approach. Specifically, the multi-age class represents a developmental context when the teachers consider: (1) differences between children as a resource for all, (2) problem-solving as an important learning strategy both for themselves and for the children, and (3) flexibility, planning, and continuous communication as essential skills for their educational work (Aina, 2001; Edwards et al., 2009).

Research shows that this educational approach adopted in the multi-age class context helps to improve teachers’ perception of self-efficacy and the perception of their well-being in the workplace (Cook et al., 2016).

Based on these reflections and on the training experience of the authors as consultants in the field of the Early Childcare and Education Centers, an interesting research question arises: could the reflective practice in the work group enhance the teachers’ perceived well-being?

Specifically, encouraged by the results of some previous studies (Karademas, 2006; Critchley and Gibbs, 2012; Zinsser and Zinsser, 2016; Venturelli and Cigala, 2017), we have identified an important research question: there are some relationships between the systematic reflective practice on some critical aspects of daily education strategies in class and some dimensions of teachers’ perceived well-being. These include: the sense of belonging (Roffey, 2012), self-efficacy (Critchley and Gibbs, 2012), and agency (Hadar and Benish-Weisman, 2019). This specific relationship finds significance in the fact that the reflective practice adopted by a work group in a systematic and continuous way allows the teachers, on the one hand, to feel more actively involved with respect to work processes in recognizing their specific role, and on the other hand, the reflection within the group allows the teachers not to perceive themselves as isolated individuals facing difficulties, but rather as a “thinking group” that guarantees protection and safety. Consequently, the reflective practice also enhances a sense of competence in the teachers in identifying solutions and directions for a possible improvement in work practices.

Starting from this research question, this study aimed to verify the efficacy of longitudinal training for the purpose of advancing the perceived well-being of early childhood teachers of multi-age groupings (18–54 months).

In particular, the aim of the present study was to investigate, with a qualitative methodology, the possible changes that occur in the perceived well-being of teachers in the course of a training path focused on reflective practice, which is a method that promotes spaces for discussion and reflection among teachers on work-related practices (Schon and DeSanctis, 2011; Pollard, 2014). More specifically, in this study teachers were offered a training course that hinged mainly on those concrete aspects, inherent to work practices, considered by the teachers themselves to be critical and problematic, with respect to which they felt the need to identify new ways to manage them and to forge new directions of work (Venturelli and Cigala, 2017).

## Materials and Methods

### Participants

Eight teachers and one pedagogical coordinator of an Italian Early Childhood Education and Care Center took part in the study. All the participants were females, of Italian origin, aged between 28 and 55 (average age: 43) with at least 5 years’ work experience and belonging to middle socioeconomic status families.

Four teachers belonged to the “young” children class made up of 20 children (from 18 months to 3 years). Four teachers belonged to the “big” children class, made up of 25 children (from 30 months to 5 years). The two classes were adjacent and placed in the same building, a nursery school building hosting a total of four classes. The training course is part of a three-year course which involved the participation of other schools. The training lasted 10 months, from September to June, and was conducted by two trainers, experts in the field of early childhood education.

Prior to the data collection, the parents’ and the teachers’ informed written consent was obtained, in compliance with the ethical norms defined by the *American Psychological Association.*

### Training Method: Reflective Practice

The training methodology, already used in other similar paths (see Venturelli and Cigala, 2017), provided for different recursive phases:

1. Emergent theme (October);
2. Observational reports (T1 time: November);
3. Reflections on actions (T1 time: December–January);
4. Re-design (T1 time February);
5. Observational reports (T2 time: March);
6. Reflections on actions (T2 time: April–May);
7. Re-design (T2 time: June)

*Emergent theme*: all of the teachers and the coordinator identified an emerging theme through a meeting in which a sharing of and a negotiation between the different teachers’ points of view takes place. The emergent themes refer to certain aspects that concern the educational practices and that the work group considers to be critical, salient, and significant at that particular time. In a subsequent meeting the identified emergent theme was shared with the trainers that contributed to defining it in detail. These meetings took place in October.

*Observational reports*: all the teachers, the pedagogical coordinator, and one trainer carried out two observations focused on the emergent theme, and subsequently they had to draw up a written protocol (observational report) of each of these observations. The observations took place at two different times: T1 time at the beginning of the training (in November) and T2 time in the second part of training (in March). In each period, 10 observational reports were drafted for a total of 20 reports. The observational reports were shared among all teachers.

*Reflections on actions*: these meetings, which involved all the participants, were aimed at reflecting on the observed practices and at sharing the different points of view and meanings that emerged from the observations. These meetings followed the observation phases. Six reflections in action sessions were conducted, divided into two periods: three meetings were realized in the first part of the training after the first 10 observations (T1 time December–January) and the other three meetings took place in the final part of the training after the second observation period (T2 time April–May). All the meetings were video-recorded and transcribed verbatim by one of the two trainers (*descriptive reports*).

*Re-designing*: in the light of the reflections that emerged in previous meetings, the teachers were invited to meet in the colleague group in order to identify and explore alternative practices and re-design new ways of acting with respect to the topic considered critical (emerging topic). The re-design phases occurred in two periods: T1 time in February and T2 time in June; in T2 time in particular the redesign session was addressed to the future work of the teachers.

### Narrative Material Collected

The narrative material that documents the training process consists of the *observational reports of the specific activities* observed (no. 20) and the *descriptive reports of reflective meetings* (no. 6). As we explained before, these protocols are collected in the course of the training, in particular in two specific periods (T1 and T2).

The observational reports of the specific activities observed were drafted by all eight teachers, by the coordinator, and by a trainer. Each actor made two observations at different times, in November (T1) and in March (T2). The task given to the teachers was to describe what had been observed as objectively as possible; it should be pointed out that these teachers often used observational reports as a working method, so this request was very understandable for them.

The *descriptive reports of reflective meeting* concern the reports of the reflection meetings held in the presence of all the protagonists of the path, coordinator, teachers, and trainers and were carried out in two different periods of time along the training (T1: December–January and T2: in April–May). The second trainer drafted these reports and they consisted of verbatim transcriptions of the video-recording of the reflection meetings.

Two different qualitative analyzes were performed on the material collected:

1. A narrative analysis of the 20 observational reports was carried out in order to point out the processes that had unfolded in the course of the training.
2. A content analysis of the six descriptive reports of the reflective meetings was carried out in order to highlight the change in the perception of teachers’ well-being over time: from the beginning of the course (T1: December–January) to the end of the course (T2: April–May).

### Process Analysis of Reflective Practice

#### Definition of the Emerging Theme

During this moment, the teachers were invited to reflect, and to propose an aspect that they experienced as being particularly critical on which they would have liked to turn their attention, and therefore on which to work within a specific path with the help of the trainers. The critical theme shared by the group was that of the *sleeping and relaxation moment* in the multi-age classroom. That is to say a moment shared by the two classes, small and big children, which provides for the collaboration by the teachers of the two classes and a common management of the space.

In particular, the analysis of the situation shows that, after a meal, both classes are offered a moment of relaxation, which takes place in a dedicated room equipped with mattresses or cots. Not all the children sleep (especially in the 3–6 age group) or at least not all at the same time, so in parallel an educational activity/play is proposed for children who stay awake. In this specific situation, the teachers’ working group and the pedagogical coordinator, for about a year, started an inter-class project, focused on the creation of an educational space that can welcome both big and small children in a sharing of spaces and educational resources.

From the exchange of teachers’ views, it emerged that this interclass sleeping and relaxation moment was recognized by teachers as a highly complex situation and was perceived as very tiring and difficult, both for the division of roles between teachers and for the organization of space. In the next meeting, the teachers shared with the trainers our analysis of the focused critical aspects; the discussion of different points of view, internal to the educational system (the teachers) and from outside the system (the trainers), enabled a more detailed analysis of the moment. In particular, the most critical aspect that emerged from the discussion was that the teachers in the *interclass sleeping and relaxation moment* having to take into consideration the different needs of children of different ages, who belong to two different classes that work separately during the rest of the day.

Finally, by means of the brainstorming technique, the group identified some questions to be highlighted through the observations, in order to improve their understanding of this moment and to better organize it: *What are the real needs of different children? How do we respond to all the different needs of children at that time? What is the role of the different adults present? How should it be coordinated in a more functional way?*

#### Observation and Reflections on Actions in Time T1

After identifying the emerging theme, the teachers, the pedagogical coordinator, and the trainer carried out the observations focused on the *sleeping and relaxation moment in the multi-age classroom*. In the subsequent reflective meetings, the trainers proposed three *key aspects* that emerged from the observations and that seemed particularly significant in relation to the critical points highlighted by the teachers: *the children’s needs*, *the adult’s role*, and *the intergroup system.*

##### The Children’s Needs

From the teachers’ observations, different needs emerged that the children expressed in the multi-age classroom at the sleeping and relaxation moment, such as: the need to sleep and relax in different ways, the need to play, and the need to share some stories and some readings with the adult. These needs were very different from each other, with major differences especially identified between small children (18 months-3 years) and the bigger ones (3–5 years).

##### Role of Adult

The analysis of observation through the lens of the adult’s role allowed us to highlight different aspects. On the one hand, many teachers’ movements appeared very dispersive and without a clear objective. Moreover, from the observational reports, it was highlighted that the teachers were an effective reference only for the children of their class, by whom they were recognized and to whom they proposed themselves as such. They did not represent a real point of reference for all the children in the class. Therefore, each teacher represented a resource for the other class only potentially but not at the level of practice. There was not a real availability of teachers for the whole inter-group. Finally, from the analysis of the observational report a good and consolidated coordination among the teachers of each class emerged.

##### Interclass System

As regards the lens of the interclass system, the analysis of the observational reports pointed out how the two classes, while occupying the same physical place, did not really seem integrated with each other. There were few contacts between the big and small children of the two classes, the teachers did not seem to perceive themselves as resources for the whole group, and there were many difficulties in fulfilling the different needs of big and small children, such as the need to relax and sleep, and the need to play and share.

A level of practice, and a clear and unique interclass project did not emerge; from the reports, it was noted that the two groups, although sharing the same physical space, seemed to be two different contiguous but unintegrated subsystems, with different times and routines.

Even the spatial organization of the room did not facilitate relationships between children, between adults, and between adults and children from different classes.

#### Re-design

In this phase of the training, the teachers were engaged in the re-design of the sleeping and relaxation moment of multi-age classroom starting from the reflections that emerged during the previous meetings. In particular, the trainers asked the group of teachers to *design*, *implement*, and *document* an interclass project linked to the sleeping and relaxation moment of the multi-age group, that contained a design shared and co-constructed by the teachers of both classes and the documentation of the project implementation.

Starting from this proposed task and from the new awareness acquired following the reflections shared in the previous meetings, the teachers agreed to work together to make the sleeping and relaxation place more familiar and more beautiful for all children and teachers.

The goal of the project was to allow all the teachers and all the children to experience this moment and the environment dedicated to this moment as being more familiar, so that everyone could feel a greater sense of belonging.

To do this, the teachers devised and planned several moments of inter-group activities that would involve all the children and all the teachers.

In particular, the project developed by the teachers included an initial circle time conversation in small groups with the children in order to highlight their experiences, thoughts, feelings about the sleeping and relaxation moment in the multi-age classroom, and their suggestions to improve it.

Two workshops then followed in small multi-age groups proposed to children in order to carry out some proposals to improve the spaces that had emerged in the conversation.

In a workshop, a structured activity was carried out for the construction of airplanes and stars, as proposed by the children themselves in the circle time; in the second workshop, the children were engaged in sky painting and in the graphic reproduction of nocturnal animals.

The children and teachers of both classes were involved in each of the proposed activities.

Finally, the multi-age sleeping area was used and furnished with new materials produced by children.

#### Observation and Reflections on Actions in Time T2

From the observational reports of the second period of the training, in the sleeping and relaxation moment, we highlight multiple relationships between big and small children of the two different classes and several occasions of shared knowledge, just as it was observed that the teachers of the different classes started to coordinate among them.

Moreover, the analysis of the observational reports showed how everyone occupied the space indiscriminately. In working together for explicitly shared common goals, the class of *big* children and the class of *small* children began to integrate within a single system.

The re-design phase at the end of the training involved a general planning of the multi-age group project to be carried out for the following year based on the new methodologies and awareness acquired during the training.

## Results

### The Perception of Teachers’ Well-Being

From the content analysis of the *descriptive report* of reflective meetings (*Reflections on actions*), the perception of well-being on the part of the teachers was detected. In particular, the aim of this qualitative analysis was to observe the perception of teachers’ well-being over time, verifying any changes that occurred in the course of the training.

The conceptual model underlying this qualitative analysis, as previously described, includes the idea that the longitudinal and systematic reflective practice that had engaged the teachers’ work group could improve the perceived well-being in some dimensions, such as: *sense of belonging*, *self-efficacy*, and *agency*.

As discussed above in the introduction, the *sense of belonging* refers to the perception of being part of a community/group, of feeling acknowledged, valued and included, and to have a precise role within the system, a role that is recognized by others (Rowe et al., 2007; Wike and Fraser, 2009; Skaalvik and Skaalvik, 2010; Roffey, 2012).

The dimension of *self-efficacy* refers to one’s self-perception as being capable of performing one’s work well and achieving the set goals, and the feeling of personal satisfaction linked to work (Karademas, 2006; Critchley and Gibbs, 2012; Kim and Yang, 2016).

The dimension of the *agency* refers, instead, to the perception of having a capacity for proactive and autonomous actions. In particular, teachers with agentic capacity feel that they have an active role within their community/work group and that they are able to participate in organizational decision-making (Wilson and Deaney, 2010; Priestley et al., 2013; Sanglim and Sungeun, 2016; Hadar and Benish-Weisman, 2019).

In accordance with some contributions from the literature (Karademas, 2006; Roffey, 2012; Hadar and Benish-Weisman, 2019) for each of these dimensions, some specific indicators of teacher well-being have been identified. Two non-independent scholars who did not know the specific hypothesis of the study, but who were experts in the research field on the topic of well-being, were engaged in order to identify from the descriptive reports some specific indicators for each of the target dimensions of well-being. Through a preliminary content analysis of the descriptive reports, the scholars individuated different indicators, each indicator being specific for a dimension of well-being, and contributed to the description of only one dimension, so that the differentiation of the three dimensions was guaranteed. This analysis consisted of a systematic and repeated reading of the descriptive reports and of a progressive identification of the content aspects that could identify the teachers’ agency, sense of belonging, and self-efficacy. The indices individuated through this method, albeit based on previous studies (Karademas, 2006; Roffey, 2012; Sanglim and Sungeun, 2016; Hadar and Benish-Weisman, 2019), appear quite new and original in the research field on teachers’ well-being.

The indicators used for each teachers’ well-being dimension are presented below.

#### Sense of Belonging

1. Use of singular/plural form
2. References to the colleagues of the class/colleagues of the other class
3. References to the children of the class/children of the other class
4. References to the whole work group
5. References to whole multi-age groups of children
6. Reference to positive emotions to stay in the multi-age classroom/negative emotions to stay in the multi-age classroom
7. Arrangement of teachers in the space during the reflective meetings

#### Self-Efficacy

1. References to the teachers’ competence-ability-skills/teachers’ incompetence
2. References to goals achieved with children/failures with children
3. References to certainty/uncertainty at work
4. References to positive emotional state (satisfaction) linked to self/negative emotional state (dissatisfaction) linked to self
5. References to teachers’ autonomy
6. References to children’s positive emotions and to their positive motivations to learning/to children’s negative emotions and to their scarce motivations to learning

#### Agency

1. References to teachers’ participation in organizational decision-making/no teachers’ participation in organizational decision-making
2. References to one’s role in the work group
3. References to colleagues’ role in the work group
4. References to co-design of the practices and of the space
5. References to re-design of the practices and of the space
6. Reference to positive changes
7. Number of teachers taking part in the reflective meetings with an active role

Following the identification of the well-being indicators, each of the six descriptive reports was systematically coded by two independent observers, reaching a satisfactory agreement index (*K* mean = 0.85). The judges who proceeded with the coding of the reports were unaware of the aims of the study and did not participate in the path with the teachers. The frequencies of the indicators for each dimension are shown in Table 1.

**Table 1:** Number of references in teachers’ descriptive reports at T1 time and T2 time.

Dimensions	Indicators	T1 no. of references	T2 no. of references
Sense of Belonging	Use of singular form	2	3
	Use of plural form	0	14
	References to the colleagues of the class	15	8
	Reference to colleagues of the other class	1	16
	References to the children of the class	6	6
	References to children of the other class	2	12
	References to the whole work group	1	8
	References to small multi-age groups of children	2	6
	Reference to positive emotions to stay in the multi-age classroom	2	9
	Reference to negative emotions to stay in the multi-age classroom	10	0
	Arrangement of teachers in the space during the reflective meetings	Two classes are separated	Independently from two classes
Self-Efficacy	References to the teachers’ competence ability-skills	0	10
	References to the teachers’ incompetence	6	1
	References to goals achieved with children	0	9
	References to failures with children	6	0
	References to certainty at work	2	7
	References to uncertainty at work	9	1
	References to positive emotional state (satisfaction) linked to self	1	18
	References to negative emotional state (dissatisfaction) linked to self	5	0
	References to teachers’ autonomy	0	6
	References to children’s positive emotions and to their positive motivations to learning	1	6
	References to children’s negative emotions and to their scarce motivations to learning	5	0
Agency	References to teachers’ participations in organizational decision-making	1	12
	References to no teachers’ no participation in organizational decision-making	4	1
	References to one’s role in the work group	0	9
	References to colleagues’ role in the work group	0	7
	References to co-design of the practices and of the space	0	10
	References to re-design of the practices and of the space	0	15
	Reference to positive changes	4	18
	Number of teachers taking part in the reflective meetings with an active role	5 out of 9	9 out of 9

#### Sense of Belonging

Regarding the aspect of the *sense of belonging* during T1 time (at the beginning of the training), the teachers were arranged in the meeting room space separately, on one side were the teachers of the first class and on the other the teachers of the second class, with the coordinator located in the middle. As shown in Table 1, the teachers used the singular form referring to both themselves and to their class (no. 25 references). In descriptive reports, no reference was made to colleagues of the other class, except in one case, but in all the remaining situations they referred only to colleagues (no. 15 references) and children in their class (no. 6 references). The teachers reported negative emotions with respect to the inter-class context, especially discomfort and a sense of estrangement (no. 10 references).

When I’m in the sleeping room I wait for my class colleague…

Our children have different habits from those in the other class.

When we are in the sleeping room, we feel we are guests of the class of younger children.

I do not quite feel I am myself in the multi-age classroom.

I do not feel this space to be mine.

The reference to the whole interclass work group was never present except in the initial statements referring to the general aim of the project. The teachers stated explicitly that there are no moments of encounter between the different classes.

There is little knowledge between us although we have been working in the same complex for 3 years.

In time T2, during the last meetings, the dimension of sense of belonging to the work group seemed to be characterized differently. First, the teachers occupied the space of the meeting room independently from it belonging to the first or second class. In regard to the use of the singular/plural form, at this point in the training, the teachers usually adopted the plural form (no. 14 references) and referred to the children (no. 12 references) and to the colleagues (no. 16 references) regardless of belonging to one or the other class. Moreover, the teachers reported positive emotions in regard to the inter-class context, especially spontaneity and ease (no. 9 references).

As teachers we decided to…

We confronted ourselves with colleagues in the other class…

We like doing things together.

We have learned to do things together.

No children of the “big” class or children of the “small” class, but just children!

I felt free as an adult to address all children.

We are more spontaneous and relaxed.

Furthermore, there are several references to the whole work group (no. 8 references) and to the small multi-age groups of children (no. 6 references).

We have co-constructed a new way of working and approaching each other through mutual knowledge and moments of sharing.

A work project has started for adults. The path began with mental availability to each other, fruitful collaboration, and sharing.

The first step in the change is the sharing between adults.

In general, the distribution of the frequencies of indicators showed that at the beginning of the training path the teachers perceived themselves as “individual practitioners”; the work group dimension did not emerge. Moreover, the analysis of the reports in time T1 showed how the interclass moment was perceived as being very difficult to handle and as being connoted by feelings of discomfort and a sense of estrangement.

Over the course of the training, a greater sense of belonging was observed, evidenced by the increased number of references to a whole work group, references to whole multi-age groups of children, and the use of plural form, such as “we.” Moreover, the teachers in the last reflective meetings reported several positive emotions in regards to staying in the multi-age classroom.

#### Self-Efficacy

With regard to the dimension of the self-efficacy, in T1 time, during the first meetings, from the descriptive reports, no references emerged to teachers’ competence-ability-skills, as well as no references to goals achieved with children.

Specifically, as shown in Table 1, the multi-age classroom was viewed with concern and negativity (no. 5 references). Many phrases and terms used by teachers indicated uncertainty (no. 9 references), and failure (no. 6 references) regarding the multi-age group of children. Many uncertainties were related to the risk of losing something positive that had already been built with and for children within the classes.

We do not want to lose the work that we have done in class with our children.

I do not know if we will be able to work in the interclass, the children are very different.

I seem unable to pay enough attention to each child.

Moreover, from the descriptive reports in the T1, references to negative emotional states of the children (no. 5 references) emerged.

Even the children seem a little confused, disoriented.

Children do not feel comfortable.

Regarding the children, they are described solely within their class, and only one reference to the children’s competence was reported. Moreover, the children were not perceived by the teachers as part of a unique interclass group.

In T2 time, during the final meetings, the descriptive reports revealed several references to teacher competence (no. 10 references), autonomy (no. 6 references), and certainty (no. 7 references) concerning the multi-age classroom. Contrariwise, the references to failure, or negative emotional states were absent. In these descriptive reports, the positive emotional states were prevalent (no. 18 references). Finally, in T2 more references to children’s positive emotions and children’s motivations to learning (no. 6 references) and achieved goals (no. 9 references) were highlighted.

It was important to recognize that children have different needs, but also to understand that we can respond to these different needs not as single adults, but as a system of adults.

Children perceive the harmony among the adults.

Children now feel more comfortable in the interclass space, and I also feel more open and available toward my colleagues of the other class.

Children are very motivated to learn new things from older partners.

Older children are learning to take care of the younger ones.

I believe we are offering children new learning opportunities.

The analysis of frequencies of the indicators revealed an important change in the teachers’ perception of themselves in the interclass system. In particular, the teachers, at the end of the training course, expressed a greater sense of competence and certainty in the management of the multi-age class. In the course of the training, the teachers’ concerns and reticence decreased and gradually a greater sense of work satisfaction emerged. In parallel, the data also showed a change in the teachers’ representation of children, who were perceived more positively, as motivated, competent, and oriented toward achieving their specific learning goals.

#### Agency

As regards the dimension of *agency*, at the T1, from the descriptive reports, no references emerged to the design or co-design of activities (Table 1). Furthermore, during the meetings at the initial period of the training, only five teachers out of nine took part in the discussion to express their opinion; in addition, the teachers often said that they agreed with what had already been reported by colleagues in their class, to indicate a strong belonging to one’s class group.

Finally, teachers reported that they did not recognize themselves as having a clear role in the management of the mixed group and not knowing how it could be possible to change the usual methods (no. 4 references); just as they believe that they do not think it is their task to introduce changes, they do not think they can suggest changes. (no. 4 references).

But what can we do? We are only teachers, those who have to decide do not are us.

In my opinion, it is difficult to introduce changes in this situation.

At T2 time, all the teachers took part in the discussions spontaneously (nine out of nine). In the descriptive reports, it was possible to note several expressions that refer to co-design (no. 10 references), and to the re-design (no. 15 references) of space practices and some references to teachers’ organizational decision-making (no. 12 references). Moreover, the teachers often referred to the individual and colleagues’ role in the work group (no. 16 references) and to potential changes that could improve the management of the multi-age classroom (no. 18 references).

This is the first time that we have designed something all together.

The training structured into repeating phases helped us a lot, making us feel more involved.

Even the children played an active role in the activities, they felt they were protagonists!

I felt I had do “my bit” for the co-construction of the project.

The relaxation room has really changed, now it is more beautiful and functional.

I believe that all of us teachers could really be protagonists of this project.

This method of working as a group could also serve to design on other aspects in the future.

The frequencies of the indicators revealed a modification, from the T1 time to the T2, in the teachers’ perception of their agentic capacity.

As shown in Table 1, in T2 time a larger number of teachers got involved in the training activities, actively contributing to reflection. Moreover, during the training a greater teacher awareness of their own role and the role of colleagues in the management of the interclass emerged. An orientation to the future also emerged: the teachers referred to possibilities of positive changes in the management of the interclass moment, implemented through the co-design and re-design of the practices and of the spaces.

## Discussion

Several scholars have pointed out that that teachers’ well-being has been mostly conceptualized in negative terms (i.e., as stress, psychological and physical health problems, and burnout) and have suggested a reconceptualization of this construct that includes positive indicators of teacher functioning, namely teaching self-efficacy, a sense of belonging to a community, and agency (Roffey, 2012; Renshaw et al., 2015).

Starting from these premises, some interesting studies that share a processual and systemic idea of teachers’ well-being have recently been carried out. Some of these aimed to study how, and by means of which processes, directors and teachers can co-construct workplaces in which the individuals can experience professional well-being (Dollard and Bakker, 2010; Zinsser and Zinsser, 2016). Other studies have instead proposed, not so much simply to reduce stress and burnout, but rather to promote positive dimensions of teachers’ well-being in some specific school contexts (Critchley and Gibbs, 2012; Roffey, 2012; Cook et al., 2016).

Within this framework, our study aimed to verify the efficacy of longitudinal training with the method of reflective practice for the purpose of advancing the perceived well-being of the early childhood teachers of multi-age groupings (18–54 months).

The results of the study make it possible to highlight which important changes in teachers’ perception occurred during the training with respect to some relevant dimensions of their working well-being. In particular, from the analysis of observational and descriptive reports, it emerges how teachers during training increased their perception of self-efficacy in the multi-age classroom. In fact, they reported a greater sense of effectiveness in their educational actions, both in terms of their own practices and the functional and adaptive behaviors of children. In support of this, from the beginning to the end of the training, we noticed a decrease in the uncertainties and concerns of teachers in respect to the multi-age classroom and an increase in positive emotions of satisfaction in the management of this moment (Karademas, 2006; Critchley and Gibbs, 2012; Huang et al., 2019).

During the training, moreover, there is also a development of the sense of belonging in terms of the perception of being part of a community/group; in fact, at the end of training, teachers referred more frequently to teachers’ whole group and to multi-age groups of children as compared with the start. Furthermore, in the final part of the training, the teachers tended to use plural forms more in their interventions in the reflective groups, and they reported positive emotional states relating to stay in the multi-age classroom (Rowe et al., 2007; Dollard and Bakker, 2010; Skaalvik and Skaalvik, 2010).

Finally, the perception of the teachers’ agentic ability was fostered by the training, in the sense that the teachers in the second part of training often described themselves as capable of proactive and autonomous actions in the management of the multi-age classroom. Also, they felt capable of participating in the organizational decision-making and to co-design the educational practices to manage the new interclass context (Wilson and Deaney, 2010; Buchanan and Bardi, 2015; Hadar and Benish-Weisman, 2019).

We believe these modifications can be attributed to some specific aspects of the proposed training. On the one hand, the method of reflective practice employed in the training allows all the teachers to experiment with an active and proactive role enabling each of them to take part in the decision-making processes in the different phases of the path. Furthermore, the training method allows the teachers to perceive themselves in their educational function, not so much as individuals, but rather as belonging to a group of colleagues, with whom it is necessary to share and negotiate strategies and educational projects.

Another important aspect of the method adopted in the course of training was the observation of the daily practices implemented in the school (Pollard, 2014). We assume that this methodological aspect has promoted in the group a sharing of new meanings and thoughts not so much on generic themes, as often happens in teachers’ training, but rather on everyday situations, behaviors, and practices. In this sense, the reflective meetings represent for the workgroup a “place” in which to understand and re-signify the usual and daily practices. (Schon and DeSanctis, 2011).

The training has given the teachers the opportunity to experience having time together, it has created a clear and step-based working context, which has been repeated over time, in which the teachers could trust each other, and in which they felt that their experience and professionalism were recognized. In this sense, we consider that even the aspect of the step-by-step approach in a sufficiently dilated time was a relevant factor for the promotion of a real change in the perception of teachers’ well-being.

Finally, in the training, it was possible for teachers to take into account the trainers’ point of view, which, as being external to the school system, allows them to broaden the perspective and to recognize new aspects and new meanings concerning the work practices.

We retain that by means of the training, the group of teachers and the work environment in general took on characteristics of greater psychological security for teachers. In fact, in a context that is perceived as psychologically secure, it is possible for teachers to engage in changes in their consolidated work practices, separate from them, and to start exploring new methods. Training of this type has been conducted with different work groups in several ECEC centers and has shown very similar results (Venturelli and Cigala, 2017).

In the light of these results, the present study could contribute to the advancement of knowledge concerning the well-being construct, both from the research point of view and applicative point of view.

With reference to the first point, we argue that the present study, in accordance with other previous research studies (Dollard and Bakker, 2010; Critchley and Gibbs, 2012; Roffey, 2012; Cook et al., 2016; Zinsser and Zinsser, 2016), could provide evidence with respect to the fact that the well-being construct is strongly influenced by systemic-relational factors. The variables that intervene in the definition of well-being cannot be read as rigidly distinguished into “personal variables” and “contextual variables,” but rather as complex variables that see the two dimensions (i.e., personal and contextual) interact together in the different daily processes that are played out in the working context. As claimed by several authors, well-being is socially and culturally constructed, rooted in a particular time and place (White, 2010; Atkinson, 2013). Therefore, for a deep knowledge of the nature of well-being, it is not enough to juxtapose descriptive variables of individuals and descriptive variables of the context, but instead we need to find methodologies and tools that allow us to detect the interdependence between these levels and how these complex relationships of interdependence vary over time.

With respect to the applicative relevance of the study, the results seem to highlight the effectiveness of the training, and therefore encourage the implementation in the contexts of the school of training courses that have some specific characteristics: they are longitudinal and systematic, focused on some critical aspects highlighted by the group of teachers and not proposed from the outside, and addressed to a small group of teachers, in order to really be able to create a real space of involvement and participation. The reflective practice, for the reasons illustrated above, appears to be a useful method, especially if it is preceded and followed by the observations in the work context, focused on the emergent critical aspects.

Starting from the results of this study and from the other research studies (Cook et al., 2016; Zinsser and Zinsser, 2016), we argue that for a true promotion and care of the teachers’ well-being it would be necessary, not so much for the occasional implementation of pathways, but instead to adopt a reflective working method, like the one proposed in the present study, as a standard practice. We believe that this is the direction in which the school should go in order to make teachers develop an idea of group and teamwork that allows them to feel more confident and competent, and therefore less vulnerable and prone to developing demotivation and burnout (Huang et al., 2019).

Moreover, the present case study has allowed us to develop a system of indicators of some relevant dimensions of perceived well-being that can be applied in descriptive and narrative material. The set of these indicators, rather original within the panorama of studies on the topic, could represent a tool for the analysis of well-being to be used in other studies, as well as a tool to evaluate the effectiveness of interventions aimed at promoting well-being in the school context.

A limit of this study is that it would have been interesting to evaluate the perception of well-being on three investigated dimensions also by means of questionnaire self-reports or semi-structured interviews both at the beginning and at the end of the path; such data could have given the obtained results greater strength. Moreover, it could be interesting to follow the work group and verify the same dimensions of well-being a few months after the end of the training to verify the maintenance of the change.

Finally, it would be useful to replicate the study by proposing training to other groups of teachers, in order to be able to make more accurate and better grounded considerations regarding the possibility of generalizing the results with respect to the effectiveness of the intervention (Woolcock, 2013).

## Data Availability Statement

All datasets generated for this study are included in the article/supplementary material.

## Ethics Statement

Ethical review and approval was not required for the study on human participants in accordance with the local legislation and institutional requirements. The patients/participants provided their written informed consent to participate in this study.

## Author Contributions

AC and EV contributed to the design and implementation of the research, to the analysis of the results, and to the writing of the manuscript. MB contributed to the drafting of the final version of the manuscript, to the conception or re-design of the work, and to interpretation of data.

### Conflict of Interest

The authors declare that the research was conducted in the absence of any commercial or financial relationships that could be construed as a potential conflict of interest.
